# Efficacy and safety of arterial blood gas analysis via distal radial artery in children

**DOI:** 10.3389/fped.2025.1611065

**Published:** 2025-12-03

**Authors:** Ling Yang, Heng Ding, Xiaoting Zhou, Shiting Shu, Wenjie Mao, Gaojun Cai, Yidong Zhao, Yu Wan, Zhiying Huang

**Affiliations:** 1 Department of Respiratory and Critical Care Medicine, The Affiliated Changzhou Second People’s Hospital of Nanjing Medical University, Changzhou, China; 2 Department of Paediatrics, The Affiliated Changzhou Second People’s Hospital of Nanjing Medical University, Changzhou, China; 3 Department of Cardiology, Wujin Hospital Affiliated with Jiangsu University, The Wujin Clinical College of Xuzhou Medical University, Changzhou, China

**Keywords:** arterial blood gas analysis, distal radial artery, radial artery, conventional radial artery, children

## Abstract

**Objective:**

The distal transradial approach is widely accepted in adult cardiovascular interventions and invasive arterial blood pressure monitoring. However, limited evidence exists regarding the efficacy and safety of arterial blood gas analysis (ABGA) via the distal radial artery (DRA) in children.

**Methods:**

This retrospective study evaluated the efficacy and safety of arterial puncture via the DRA compared with the conventional radial artery (CRA) for ABGA in children.

**Results:**

A total of 240 children were allocated to either the DRA group (*n* = 140) or the CRA group (*n* = 100). The success rate (82.1% *vs.* 92.0%, *p* = 0.029) and one-needle success rate (75.7% *vs.* 89.0%, *p* = 0.009) were significantly lower in the DRA group. Puncture time in the DRA group was significantly longer than that in the CRA group, whereas hemostasis time was shorter in the DRA group. The incidences of incorrect venipuncture (11.4% *vs.* 1.0%, *p* = 0.002) and thumb numbness (5.7% *vs.* 0.0%, *p* = 0.022) in the DRA group were significantly higher than those in the CRA group. However, the incidence of hematoma (modified EASY Ia type) in the DRA group was significantly lower than that in the CRA group (8.6% *vs.* 50.0%, *p* < 0.001). No significant differences were observed in the incidences of hemorrhage or radial artery occlusion between the two groups.

**Conclusion:**

DRA puncture for ABGA in children appears to be a safe and practical alternative to CRA. Although first-attempt success is slightly lower and training is required, the reduced risk of hematoma and shorter hemostasis time make it a promising option for clinical use.

## Background

Arterial blood gas analysis (ABGA) is a common procedure for children with acute and critical respiratory diseases (1). It is performed at sites such as the conventional radial artery (CRA), femoral, and dorsalis pedis arteries, each with its own advantages and disadvantages. Puncture of the CRA for ABGA in children is most widely used in clinical practice due to its superficial location, high success rate, patient comfort, and short compression hemostasis time (2). However, transradial blood gas collection can lead to puncture-related complications. These include pseudoaneurysm, carpal tunnel syndrome, severe hemorrhage, and radial artery occlusion (RAO) (3–6).

In recent years, the distal radial approach has been widely adopted in adult cardiovascular interventions and invasive arterial pressure monitoring (7–9). Although the success rate of distal radial artery (DRA) puncture is lower than that of CRA puncture, it has the advantage of reducing RAO risk and hemostasis time (10). An occluded radial artery not only limits future use (11) but may also impair hand development in children. The preservation of the CRA is a critical consideration in children. This is due to their greater need for “vascular capital”, as they have a much longer lifespan than adults. However, limited research has addressed the safety and effectiveness of ABGA via DRA in children. Therefore, this study aimed to evaluate the efficacy and safety of ABGA via DRA in children at our center.

## Methods

### Study population

Children requiring ABGA for severe respiratory diseases in the Department of Paediatrics were enrolled in two time periods. In the first stage (August to September 2023), ABGA via DRA was performed. In the second stage (December 2024 to March 2025), ABGA via CRA was performed. Inclusion criteria: children aged 2–14 years scheduled for ABGA. Exclusion criteria: (1) absence of a clearly palpable radial artery; (2) puncture contraindications; (3) designated nurse not on duty; and (4) refusal to sign informed consent. This study was approved by the Ethics Committee of the Affiliated Changzhou Second People's Hospital of Nanjing Medical University [Approval number: (2023) YLJSA069]. Written informed consent was obtained from each participant and/or guardian.

### Procedure of DRA puncture

**Figure 1:**
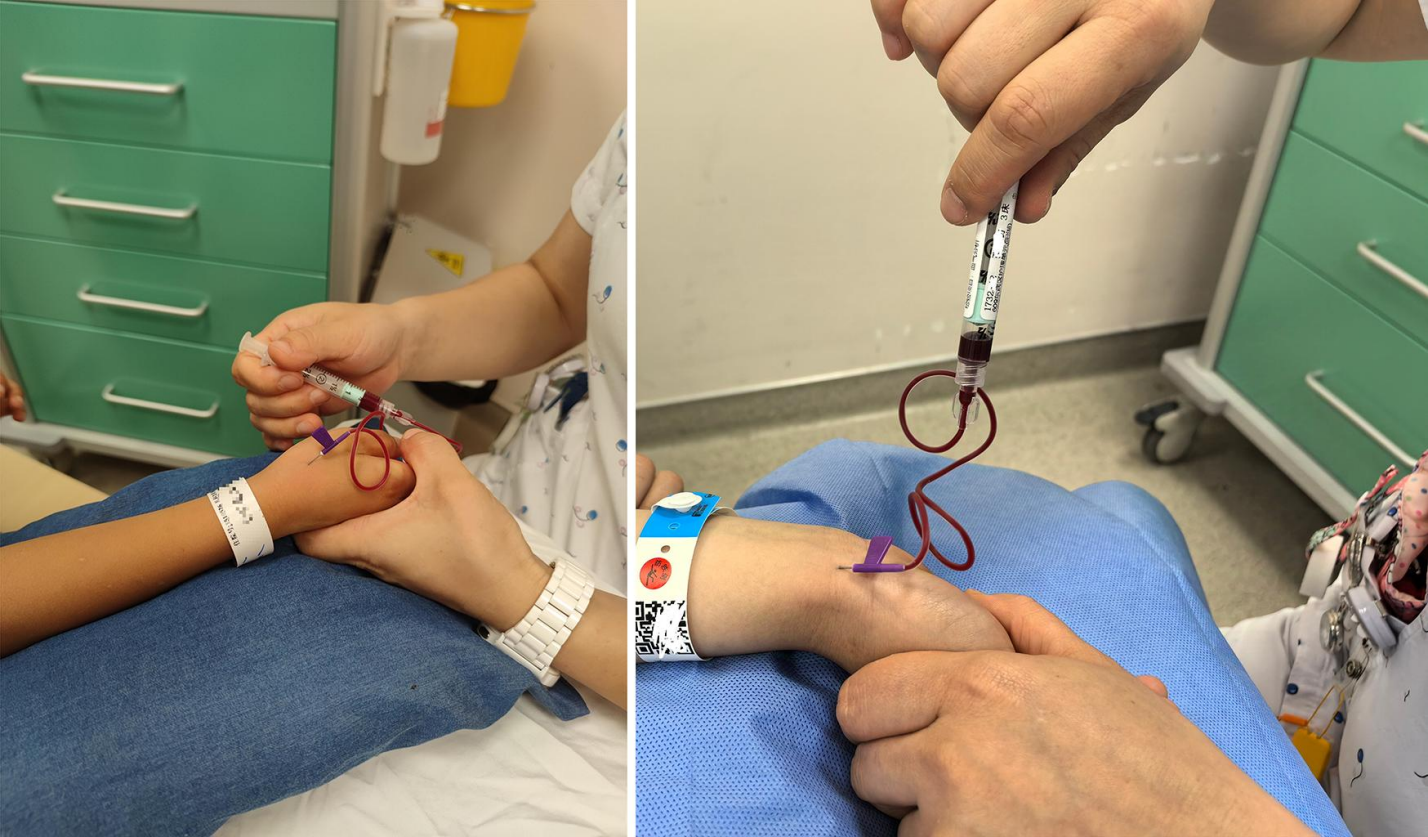
Procedure of arterial puncture [**(A)**, distal radial artery **(B)**, conventional radial artery]. After local disinfection and locating the artery pulse, the nurse held the scalp needle (0.55 × 16.2 mm) and inserted it into the AS at a 30–45° angle.

A nurse (Xiaoting Zhou) with experience in >5,000 ABGA procedures via CRA but none via DRA was assigned to perform the punctures. For DRA puncture, the child placed the hand a functional position on a small mat. After local disinfection and locating the DRA pulse, the nurse held the scalp needle (0.55 × 16.2 mm) and inserted it into the AS at a 30–45° angle (Figure 1A). After collecting blood, manual hemostasis was applied. For CRA puncture, the child's forearm was externally rotated with the palm facing upward, and the wrist slightly dorsiflexed. The operator palpated the strongest radial artery pulse near the medial side of the radial styloid process to identify the puncture site (Figure 1B). The remaining steps mirrored those of DRA puncture.

### Definition

Puncture time was defined as the interval from the end of disinfection to successful arterial puncture. Puncture success was defined as obtaining an arterial blood gas sample within 2 min. Each needle insertion represented one skin puncture (2). Bleeding was classified using the Bleeding Academic Research Consortium (BARC) criteria (12). Forearm hematoma was defined as swelling near the puncture site following blood gas sample collection and classified using the modified EASY (Early Discharge After Transradial Stenting of Coronary Arteries Study) scale, as detailed by Tsigkas G *et al*. (13).

### Statistical analysis

Statistical analysis was performed using SPSS 21.0. Categorical data were expressed as numbers (percentages) and compared using the chi-squared or Fisher's exact test. Continuous variables were reported as means ± standard deviations if normally distributed, which were tested using Kolmogorov–Smirnov method. Comparison between groups was performed using the *t*-test; Otherwise, medians (interquartile range) were reported and analyzed using the Mann–Whitney U test. Changes in DRA puncture success rates over time were assessed using a trend *P* test. To eliminate confounding factors, logistic regression model was used to investigate the independent influencing factors of the puncture success rate, adjusting for age (≥6 years old or ˂6 years old), gender (boy or girl), and BMI (≥15.09 kg/m^2^ or ˂15.09 kg/m^2^). Age and BMI were dichotomized at the median. A *P*-value < 0.05 was considered statistically significant.

## Results

### Participant characteristics

Table 1 presents the characteristics of the participants. A total of 240 participants, aged 6 (4–8) years, were included. Among them, 111 (49.3%) were male. Based on the initial puncture site, participants were classified into the DRA group (*n* = 140) and the CRA group (*n* = 100). There were no significant differences between the two groups in age, sex, height, weight, or vital signs. Platelet and glucose levels were significantly higher in the DRA group than in the CRA group, whereas body mass index (BMI) and creatinine in the DRA group were significantly lower than those in the CRA group.

**Table 1:** Comparison of the demographics and clinical characteristics between DRA and CRA groups.

Variables	Total (*n* = 240)	CRA (*n* = 100)	DRA (*n* = 140)	*t/Z/χ^2^*	*P*
Demographic					
Age, y	6 (4–8)	6 (4–9)	6 (4–8)	0.85	0.394
Boy, *n*(%)	111 (46.3)	43 (43.0)	68 (48.6)	0.73	0.393
Hight, cm	120.0 (105.0–135.0)	119.0 (105.0–141.8)	120.0 (105.0–134.8)	0.60	0.546
Weight, kg	21.0 (16.1–29.0)	21.5 (17.0–32.6)	21.0 (15.5–27.8)	1.77	0.077
BMI, kg/cm^2^	15.1 (13.9–16.5)	15.7 (14.4–17.7)	14.7 (13.6–16.4)	2.94	0.003
Vital sign					
SBP, mmHg	104.1 ± 11.5	104.4 ± 10.7	103.9 ± 12.1	0.34	0.730
DBP, mmHg	66.0 ± 8.5	66.7 ± 8.2	65.5 ± 8.7	1.13	0.260
HR, bpm	104.3 ± 15.7	105.9 ± 15.0	103.1 ± 16.1	1.33	0.183
Laboratory findings					
WBC, 10^9^/L	7.1 (5.7–9.8)	7.1 (5.7–9.6)	7.1 (5.8–9.9)	0.63	0.532
HGB, g/L	122.0 (116.0 −127.0)	123.5 (117.0–130.0)	122.0 (115.3–127.0)	1.66	0.094
PLT, 10^9^/L	299.5 (238.5–358.8)	290.5 (224.3–344.3)	308.5 (249.3–374.8)	2.38	0.017
ALT, u/L	27.0 (22.2–32.0)	27.7 (23.5–32.7)	26.2 (22.0–31.0)	1.39	0.165
AST, u/L	11.9 (9.1–14.7)	12 (9.3–16.0)	11.8 (9.0–14.0)	0.68	0.519
Cr, μmol/L	30.7 (26.8–36.2)	33.5 (29.3–38.2)	28.5 (24.7–33.4)	5.25	˂0.001
Glu, mmol/L	5.3 (4.7–6.0)	5.2 (4.6–5.6)	5.4 (4.8–6.3)	2.61	0.009

Data are presented as *n* (%), median (interquartile range), or mean ± SD; CRA, conventional radial artery; DRA, distal radial artery; BMI, body mass index; SBP, systolic blood pressure; DBP, diastolic blood pressure; HR, heart rate; WBC, white blood cell; HGB, hemoglobin; PLT, platelet; ALT, alanine aminotransferase; AST, aspartate transaminase; Cr, creatinine; Glu, glucose.

### Comparison of ABGA effectiveness between DRA and CRA groups

Right-side puncture was performed in 96.4% of cases in the DRA group and 100.0% in the CRA group (*p* > 0.05). The puncture success rate (82.1% *vs.* 92.0%, *p* = 0.029) and one-needle success rate (75.7% *vs.* 89.0%, *p* = 0.009) were significantly lower in the DRA group than in the CRA group (Table 2). Logistic regression model was used to eliminate confounding factors. After adjusting for age, gender, and BMI, the results showed that DRA was the independent influencing factors of the puncture success rate (For success rate, adjusted *p* = 0.032; For one-needle success rate, adjusted *p* = 0.014). In addition, the puncture time in the DRA group was significantly longer than that in the CRA group. However, the hemostasis time was shorter in the DRA group.

**Table 2:** Comparison of the effectiveness and safety of ABGA between DRA and CRA groups.

Variables	CRA (*n* = 100)	DRA (*n* = 140)	*Z/χ^2^*	*P*
Right side, *n*(%)	100 (100.0)	135 (96.4)	3.65	0.077
Success rate, *n*(%)	92 (92.0)	115 (82.1)	4.78	0.029
One-needle success rate, *n*(%)	89 (89.0)	106 (75.7)	6.76	0.009
Puncture time, s	4.0 (2.0–11.0)	15.5 (5.0–35.0)	6.51	˂0.001
Hemostasis time, s	60.0 (60.0–60.0)	60.0 (60.0–60.0)	2.66	0.008
Venous puncture, *n*(%)	1 (1.0)	16 (11.4)	9.64	0.002
Bleeding, *n*(%)	0 (0.0)	1 (0.7)	0.72	1.000
Hematoma, *n*(%)	50 (50.0)	12 (8.6)	52.26	˂0.001
Numbness, *n*(%)	0 (0.0)	8 (5.7)	5.91	0.022
VAS	4.0 (2.0–4.0)	4.0 (2.0–7.0)	3.91	˂0.001
DRA or RPA occlusion, *n*(%)	0 (0.0)	0 (0.0)	-	-

ABGA, arterial blood gas analysis; DRA, distal radial artery; CRA, conventional radial artery; VAS, visual analogue score.

**Figure 2:**
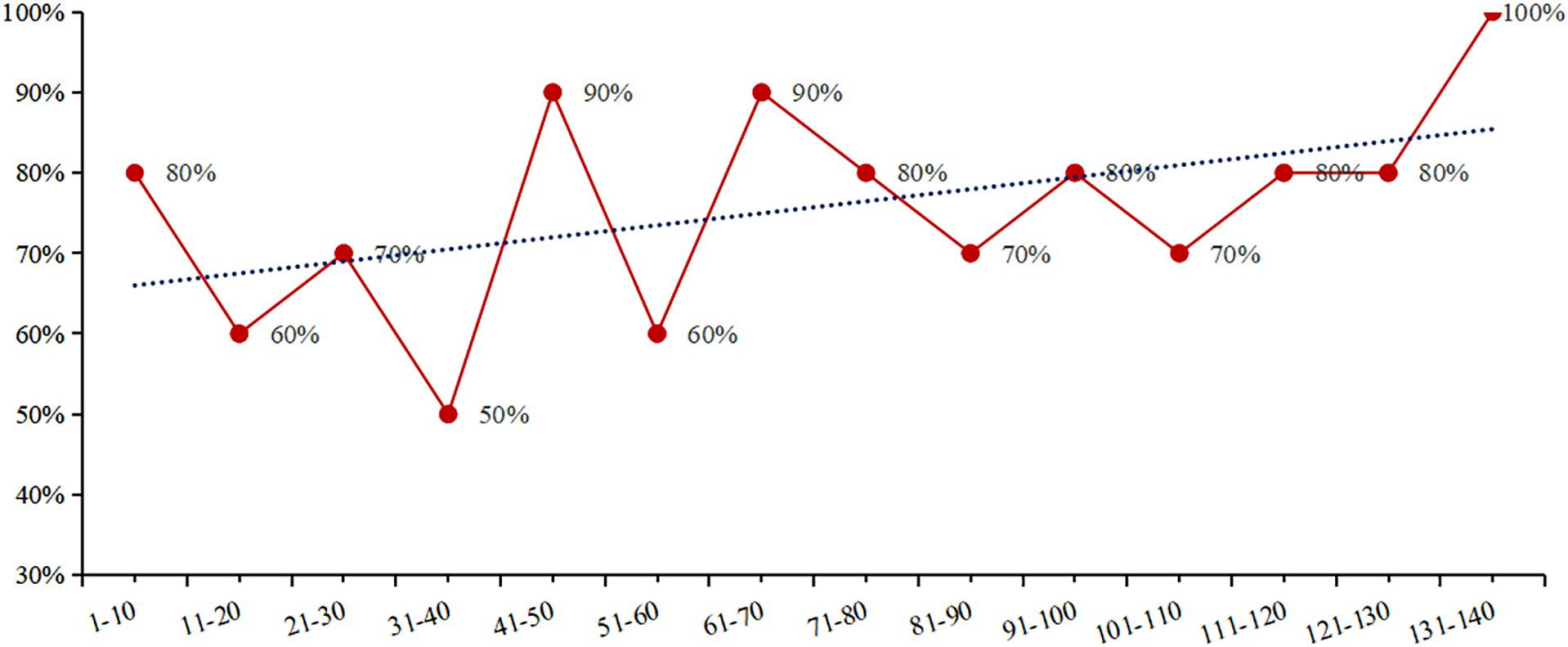
The change of success rate in DRA puncture over time.

To examine changes in DRA puncture success over time, patients were divided into 14 groups (10 per group). The success rate ranged from 50.0% to 100.0%, showing a non-significant upward trend (Figure 2).

### Comparison of the safety of ABGA between DRA and CRA groups

The incidence of incorrect venipuncture was significantly higher in the DRA group than in the CRA group (11.4% *vs.* 1.0%, *p* = 0.002). Additionally, the incidence of thumb numbness and the VAS scores were significantly higher than in the DRA group than those in the CRA group. However, hematoma (modified EASY I type) occurred in 12 cases in the DRA group, significantly fewer than in the CRA group (8.6% *vs.* 50.0%, *p* < 0.001). No significant differences were observed in the incidences of hemorrhage or RAO between the two groups.

## Discussion

To our knowledge, this is the first study to investigate the efficacy and safety of ABGA via the DRA in children. The findings suggest that DRA can serve as an alternative for arterial blood gas sampling, although its puncture success and one-needle success rates were lower than those of CRA.

The radial artery is increasingly used for arterial access during cardiovascular interventions and for blood pressure monitoring under anesthesia in children ^(^14–17). The standard ABGA site is 1–2 cm proximal to the wrist transverse striation, that is, the CRA. One-needle blood gas sampling via the CRA has a success rate of approximately 90% (6). In patients with a weak pulse or repeated failed attempts, ultrasound-guided puncture may enhance success (2). However, the radial artery is vulnerable to damage, particularly with repeated access (6). With increasing life expectancy, radial artery preservation is gaining importance, as the vessel may be reused in procedures such as interventional access, coronary artery bypass grafting, or arteriovenous fistula hemodialysis (18–20). Damaged radial arteries may compromise future use (11), prompting calls for increased protection (21, 22). The DRA, located distal to the radial styloid process, travels beneath the AS and through the first and second metacarpal spaces to form the deep palmar arch (23). Due to its superficial position and underlying bony support, the DRA is favorable for puncture and compression. Even when the radial pulse is weak, successful puncture is possible due to its anatomical course through the first and second metacarpal spaces (24, 25).

To date, the efficacy and safety of using the DRA in cardiovascular intervention and perioperative arterial blood pressure monitoring in adults have been well demonstrated (8, 9, 26–28). Studies have shown that compared with the CRA approach, the DRA approach can significantly shorten compression hemostasis time and reduce RAO and other vascular access-related complications (8, 26). However, prolonged puncture time and relatively low success rate remain disadvantages of DRA in cardiovascular intervention, largely due to the learning curve, smaller diameter, and tortuosity of the artery. In cases of failed sheath placement following successful needle puncture, the most common cause is the inability to advance the guidewire (13). A recent non-inferiority randomized controlled trial found no significant difference in first-attempt success rates for arterial blood sampling via DRA compared with CRA in adults (77.8% *vs.* 80.0%, *p* = 0.72), although the sample size was relatively small ^(^29). However, reports on the use of DRA puncture in children remain limited. In the present study, we reported preliminary experience regarding the efficacy and safety of ABGA via DRA in children. As expected, the success rate (82.1% *vs.* 92.0%) and one-needle success rate (75.7% *vs.* 89.0%) for DRA were significantly lower than those for CRA. Several factors may explain the lower success rate. First, anatomical characteristics of the DRA in children contribute to puncture difficulty. The average radial artery diameter in children has been reported as 1.39 mm in males and 1.57 mm in females, smaller than in adults and increasing gradually with age ^(^17, 30). Our previous study found that the mean DRA diameter in children was 1.97 ± 0.37 mm, increasing with age and BMI, and smaller than that of the CRA (31). A smaller vessel diameter may reduce puncture success. In addition to vessel size, low BMI was also an independent risk factor for puncture failure (32). In this study, BMI in the DRA group was lower than in the CRA group. Moreover, nurse experience with DRA puncture played an important role in success rates. Studies in cardiovascular intervention via DRA have identified a learning curve, with puncture time stabilizing after approximately 150 procedures (33). To assess whether a learning curve existed in DRA puncture, patients were divided into time-based subgroups. Although not statistically significant, a trend toward improved success over time was observed, likely due to the small sample size. It is important for operators to be familiar with the anatomy of the DRA and receive training in the puncture technique to improve the success rate.

During DRA puncture, positioning the child's hand in a functional posture may improve comfort. Additionally, the incidence of complications such as hematoma was lower in the DRA group. However, incorrect venipuncture and finger numbness were common, which may be related to the anatomy of the DAR. In the DRA group, 64% (16/25) of puncture failures resulted from incorrect venipuncture. The cephalic veins on the dorsal hand are well developed. In the AS region, the cephalic vein may overlie the radial artery, necessitating caution during puncture. The superficial branch of the radial nerve (SBRN), which innervates the dorsoradial skin and two-and-a-half fingers, also traverses the AS. Theoretically, blind puncture carries the risk of nerve injury (34). In adult, the mean horizontal and vertical distances from the DRA to the SBRN were 5.10 (2.60–7.90) mm and 2.40 (1.70–3.20) mm, respectively. The horizontal distances was less than 5.0 mm in 49% of patients (35). However, there is currently an absence of research data in the paediatric population. When compared to the CRA group, the DRA group required more puncture attempts and a longer time, which might elevate the risk of complications. These risks tend to decrease with accumulated experience. In addition, ultrasound guided puncture might reduce the complications in children. RAO in the DRA and/or CRA was not observed in this study. However, assessment was based on pulse palpation, which may underestimate true incidence. The median hemostasis time was 60 s. This short duration is a clear advantage of DRA use.

## Limitations

First, this was a small-sample observational study evaluating the efficacy and safety of ABGA via DRA in children. Retrospective study also has inherent limitation in statistical power. Prospective, multicenter, large-sample, randomized controlled studies are needed for further clarification. Second, ABGA was performed by only one nurse experienced in CRA puncture but not in DRA puncture, contributing to a relatively low initial success rate due to the learning curve, which was a key limitation of the study. However, it truly reflects the learning curve of an operator experienced in CRA puncture when adopting the DRA puncture. It is encouraging that even for operators with limited experience in DRA puncture, the success rate of puncture exceeds 80.0%. Randomized-controlled study conducted by operators with extensive experience both in DRA and CRA puncture have registered in Chinese Clinical Trial Registry (ChiCTR2500108780), which may provide evidence on whether ABGA via DRA is non-inferior to the CRA. Third, two-period design due to the off-site training of the operator, may lead to potential temporal and selection bias. However, the patients were consecutively enrolled in two periods to minimize selection bias. In addition, logistic regression model was also used to eliminate confounding factors. Fourth, no ultrasound follow-up was performed to assess arterial injury, and the long-term impact of DRA puncture remains unclear. The method of palpation may underestimate the incidence of RAO, due to the presence of palmar arch circulation. Therefore, the absence of RAO in the study should be interpreted cautiously.

## Conclusion

DRA puncture for ABGA in children appears to be a safe and practical alternative to CRA. Although first-attempt success is slightly lower and training is required, the reduced risk of hematoma and shorter hemostasis time make it a promising option for clinical use.

## Data Availability

The raw data supporting the conclusions of this article will be made available by the authors, without undue reservation.
